# MiR-1244 sensitizes the resistance of non-small cell lung cancer A549 cell to cisplatin

**DOI:** 10.1186/s12935-016-0305-6

**Published:** 2016-04-12

**Authors:** Weili Li, Wenzhe Wang, Mingjian Ding, Xiaoliang Zheng, Shenglin Ma, Xiaoju Wang

**Affiliations:** Center for Molecular Medicine, Zhejiang Academy of Medical Sciences, Hangzhou, Zhejiang People’s Republic of China; Department of Radiation Oncology, Affiliated Hangzhou Hospital of Nanjing Medical University, Hangzhou, Zhejiang People’s Republic of China; Institute of Lung Cancer, Zhejiang Academy of Medical Sciences, Hangzhou, Zhejiang People’s Republic of China

**Keywords:** microRNA, Non-small cell lung cancer, Cisplatin-resistance, Target therapy

## Abstract

**Background:**

Cisplatin (DDP)-based chemotherapy is the mainstay of first-line therapy for lung cancer. However, their efficacy is often limited by the existence or development of chemoresistance. The aim of this study was to find and investigate the function of miRNAs in cisplatin (DDP)-resistant non-small cell lung cancer (NSCLC) A549 cell.

**Methods:**

Quantitative real-time PCR assay was employed to compare the differences of miRNA expression in both cisplatin-resistant A549 (A549/DDP) cell and the parental A549 cell. The dysregulated miRNAs were then corrected by transfecting oligonucleotides into A549/DDP cells. The cellular sensitivity to cisplatin, cell apoptosis and migration were conducted by MTT, flow cytometry and cell wound healing assay, respectively.

**Results:**

Both miR-589 and miR-1244 were significantly down-regulated in A549/DDP cell compared to the parental A549, while the expression of miR-182 and miR-224 were increased in A549/DDP cell (P < 0.05). Importantly, transfection of the cisplatin-resistant cells with either miR-589 or miR-1244 resulted in an increased sensitivity to cisplatin, indicating that the dysregulated miRNA may play an important role in chemotherapy resistance in cancer cell. The rescued expression of miRNA also reduced cell invasion and increased apoptosis of A549/DDP cell.

**Conclusion:**

The study indicates a crucial role of miR-1244 in the progress of cisplatin resistance of A549. Further understanding of miR-1244-mediated signaling pathways may promote the clinical use of miR-1244 in lung cancer therapy.

## Background

Lung cancer is one of the most common malignancy worldwide [1]. Almost 85 % of lung cancer cases belong to non-small-cell lung cancer (NSCLC) [2]. Currently, chemotherapeutic agents are widely used in the treatment of lung cancer. Cisplatin (DDP), a platinum-based compound, is one of the first-line chemotherapeutic agents for the treatment of NSCLC [3, 4]. However, its efficacy is often limited by the development of chemoresistance [5, 6]. Therefore, study of the molecular mechanisms of DDP resistance will aid the clinician to oversee the resistance in advance thus improving the efficacy of lung cancer therapeutics.

microRNAs is a family of small, non-coding RNAs which function as a novel class of gene expression regulators at posttranscriptional level [7–9], thus resulting in mRNA destabilization and translational repression [9–11], a process involved in the regulation of cellular development, proliferation, differentiation, apoptosis and metabolism [9, 12–14]. Significant amount of studies showed dysregulations of miRNAs are associated with the initiation and progression of cancers [15, 16]. Different expression levels of miRNA were found in various human cancers including NSCLC [16]. Recently, the miRNA expression was observed to be linked with tumor response to chemotherapies, including cisplatin [17, 18].

In order to study the molecular mechanisms of miRNAs for the acquired DDP resistance of lung cancer cells, we firstly established a DDP-resistant lung cancer cell (A549/DDP) from the parental A549, a cisplatin sensitive line. We found that miR-589 and miR-1244 were significantly down-regulated in the A549/DDP cell line. This is interesting, as there has no published data on the roles of miR-589 and/or miR-1244 in the development of DDP-resistance of lung cancer cells. Therefore, we hypothesized that miR-589 or miR-1244 may play an important role in chemotherapy resistance in NSCLC.

## Methods

### Cell culture

The parental lung cancer A549 cell was purchased from Shanghai Institute of Cell Biology (Shanghai, China). The DDP-resistant cell line (A549/DDP) was established as previously published [19]. Briefly, DDP was added into A549 cells in the log phase at a concentration of 0.2 μg/ml and remained in the medium. After growth, the cells were split and treated again with progressively higher concentrations of DDP. During the treatment, the DDP concentration was increased to 15 μg/ml. All cell lines were cultured in Dulbecco’s modified Eagles medium (DMEM) containing 10 % fetal bovine serum (Gibco, NY, USA) in the humidified air with 5 % CO_2_ at 37 °C.

### Transfection of microRNA mimics or inhibitors

Cells seeded in a 6-well plate (2.5 × 10^5^ per well) were transfected at 50 % confluence using Lipofectamine RNAiMAX Transfection Reagent (Invitrogen, CA, USA) with Opti-MEMI (Gibco, NY, USA) according to the manufacturer’s instructions. After 24 or 48 h, the transfected cells were harvested for downstream analyses or measured for cell wound healing assay. Both of microRNA mimics, inhibitors and their negative control were purchased from Invitrogen (Invitrogen, CA, USA).

### Quantitative real-time PCR (qRT-PCR) assays

Total RNA was isolated using TRIzol reagent (Invitrogen, CA, USA) according to the manufacturer’s instructions, and A260/280 and A260/230 ratios were measured using the Nanodrop 2000 (Thermo Scientific, PA, USA). Approximately 500 ng of total RNA was converted to cDNA by First-strand cDNA synthesis kit (OriGene Technologies, MD, USA). Quantitative real-time PCR was then conducted using SYBR^®^ Green mastermix (cwbio, China) in a 7500 Fast PCR instrument (Applied Biosystems, CA, USA). All samples were run in triplicate in the 96-well reaction plates.

### MTT assay

The cells (2 × 10^3^) in the log growth phase were seeded in a 96–well plate. After overnight growth, the cells were transfected by oligonucleotides (Invitrogen, CA, USA) as described above. After 24 h of transfection, the cells were treated with different dose of cisplatin (0, 20, 40, 80, 160 µg/ml) and incubated for 48 h. A total of 20 μl of 5 mg/ml MTT reagent (Sigma-Aldrich, MO, USA) was added and incubated in the incubator for 4 h. The plate was then read in a microplate reader at a wavelength of a 490 nm reference.

### Cell wound healing assay

The cells were seeded in a 6-well plate (1 × 10^6^ per well). After confluent, a linear wound was made using a 10 μl pipette tip across the cell monolayer. The cells were grown in DMEM supplemented with 3 % FBS for additional 48 h. The cell motility was measured by photographing five random areas at the time of 0, 24 and 48 h after wounding.

### Analysis of apoptosis by flow cytometry

The annexin V-FITC Apoptosis Detection Kit (MultiSciences Biotech Co, China) was used to quantify apoptosis by flow cytometry. The cells were harvested in PBS and collected by centrifugation. The cells were then re-suspended in the binding buffer, stained with FITC and PI for 5 min and immediately analyzed by flow cytometer.

### Western blot assay

The cells were harvested and lysed in RIPA buffer in the presence of protease inhibitors (Roche, Mannheim, Germany) for 10 min at 4 °C. Protein concentration in the supernatants was quantified using the Bio-Rad Protein Assay Kit (Bio-Rad Laboratories, CA, USA). Proteins (30 μg) were separated by 15 % SDS-PAGE and then transferred to PVDF membranes (Millipore, Bedford, MA). After blocking with 5 % nonfat milk in TBST for 1 h, the blot was incubated with the primary antibodies overnight at 4 °C. After three washes (15 min each) in TBST, blots were incubated with horseradish peroxidase-conjugated secondary antibodies and developed using an ECL detection system. All images showed were from a single experiment that was a representative of at least three independent replicate experiments.

### Bioinformatics and statistical analysis

MiRNA target analysis was conducted by miRDB (http://www.mirdb.org/miRDB/), Targetscan (http://www.targetscan.org/) and CLIP-Seq. All data were presented as the mean ± SD of three or more replications, and the Student’s t test was used to test the significance between two groups. Data analysis was performed using GraphPad Prism 4.0 (Graph Pad Software, La Jolla, CA) and two-tailed P < 0.05 were considered to be significant.

## Results

### The different expression of miRNA between A549 and A549/DDP cells

**Fig. 1:**
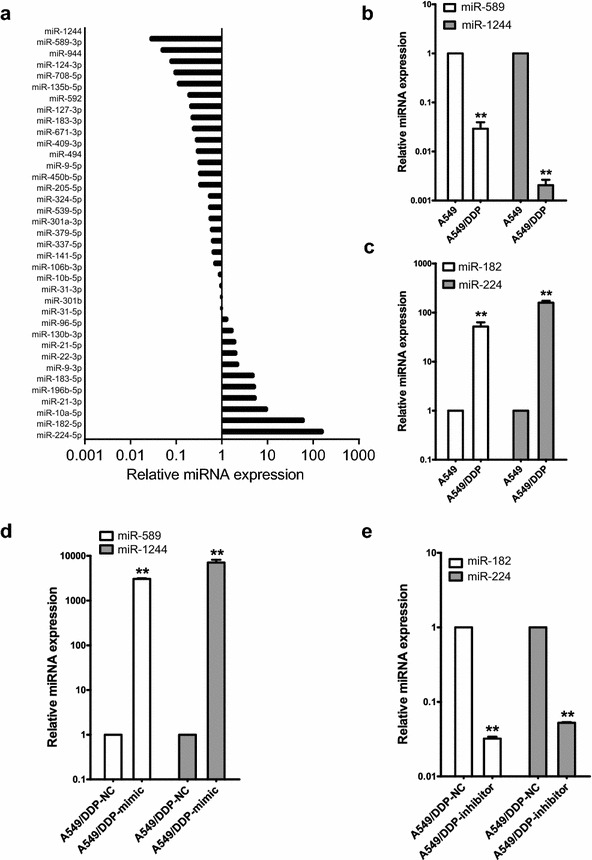
Quantitative real-time PCR analysis of the expression of microRNAs in A549 cells and A549/DDP cells. **a** The relative expressions of 37 miRNAs in A549/DDP cells. The data were normalized by their expression in A549 cells. **b**, **c**The expression levels of miR-1244, miR-589, miR-224 and miR-182. The results are the average of triplicate experiments. **d**, **e** Fold changes of miR-1244, miR-589, miR-224 or miR-182 in A549/DDP cells after 48 h of transfection with the mimics or inhibitors, respectively. The results are the average of triplicate experiments. **P < 0.05

Previously, we have identified by array analysis a set of miRNAs differentially expressed in NSCLC tissues compared to healthy controls (manuscript under review). In this study, we employed qRT-PCR assay to assess the expression level of the miRNAs in both A549 and A549/DDP cells. Among 37 miRNAs, we observed that 11 miRNAs were upregulated and other 26 miRNAs were down-regulated in A549/DDP cell line (Fig. 1a). Especially, miR-589 and miR-1244 were significantly decreased in A549/DDP cell (Fig. 1b), while miR-182 and miR-224 were increased in A549/DDP cell (Fig. 1c).

### Transfection with miR-mimics or miR-inhibitors

MiRNA mimic is a short sequence of oligonucleotides synthesized by chemical method which can enhance the functions of endogenous miRNA, while miRNA inhibitor is a modified oligonucleotide that can incorporate into the target miRNA thus attenuating its function. To further assess the effects of the dysregulated miRNA on DDP resistance of A549/DDP cell, we transfected the miRNA mimic for miR-589 or miR-1244, and the miRNA inhibitor for miR-182 or miR-224 into A549/DDP cells. As shown in Fig. 1c, the relative expression levels of miR-589 and miR-1244 are significantly increased in A549/DDP transfected by mimic miRNAs compared to the negative controls (Fig. 1d). Likewise, the expression level of miR-182 and miR-224 are significantly decreased in A549/DDP cell after transfection of miRNA inhibitors (Fig. 1e), indicating both mimic and inhibitory oligonucleotides could revert, at least in partial, the expression level of its corresponding miRNA.

### The miRNA mimic sensitizes A549/DDP cells to DDP

**Fig. 2:**
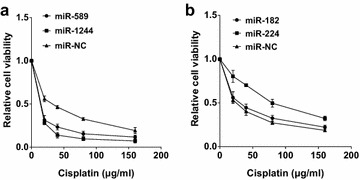
Sensitivity of A549/DDP to cisplatin by MTT assay. **a** Overexpression of miR-589 or miR-1244 attenuates the sensitivity of A549/DDP cells in response to DDP compared to A549/miR-NC cells. **b** Down-regulation of miR-182 or miR-224 has no effects on the sensitivity of A549/DDP cells to DDP. Each treatment was carried out in quadruplicate

We hypothesized that the corrected miRNA expression might sensitize A549/DDP cells to DDP. To this end, we first transfected A549/DDP cells by either miR-589 mimic or miR-1244 mimic followed by the treatment with various doses of DDP (0, 20, 40, 80 and 160 μg/ml) for 48 h. The MTT assay showed that up-regulation of miR-589 or miR-1244 led to a significant decrease in cell viability of A549/DDP in response to DDP compared to A549/DDP-NC (Fig. 2a). The IC50 value for DDP in both A549/DDP-miR-1244 (5.66 ± 2.32 μg/ml) and A549/DDP-miR-589 cell (6.53 ± 2.21 μg/ml) were significantly lower that of A549/DDP-miR-NC cell (30.1 ± 3.44 μg/ml) (P < 0.05). However, reduction of miR-182 or miR-224 has no effects on the sensitivity of A549/DDP to DDP (Fig. 2b). The IC50 in A549/DDP-anti miR-224, A549/DDP-anti miR-182 and A549/DDP-anti miR-NC cells were 83.76 ± 5.03, 28.77 ± 4.09 and 23.7 ± 2.47 μg/ml, respectively. These data suggested that up-regulation of miR-589 or miR-1244 effectively enhances the sensitivity of A549/DDP cells to DDP.

### Over-expression of miR-589 or miR-1244 induces cell apoptosis

**Fig. 3:**
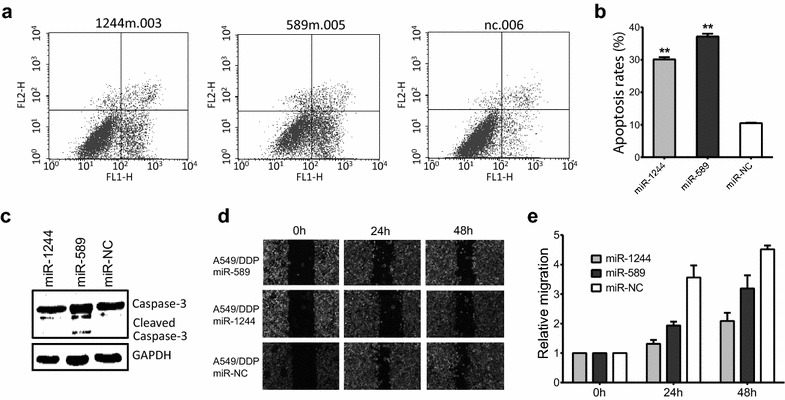
Effects of miR-1244 or miR-589 on cell apoptosis and migration in A549/DDP cells. **a**, **b** Flow cytometry analysis of apoptosis in A549/DDP-miR-1244 cells (*left*) or A549/DDP-miR-589 cells (*middle*) compared to A549/DDP-miR-NC cells (*right*). **c** Western blot analysis of caspase-3 expression in response to either miR-589 or miR-1244 transfection in A549/DDP cells. GAPDH was used as internal control. **d** The wound healing assay was performed on A549/DDP cell transfected by miR-589 or miR-1244. **e** Migration rate of each group. All experiments were performed in triplicate, and a representative image is shown (**P < 0.05)

We next analyzed the effect of miR-589 or miR-1244 by flow cytometry on apoptosis of A549/DDP cell after introduction of miR-589 or miR-1244. The apoptotic cells were increased in A549/DDP cell transfected with miR-589 or miR-1244 mimics, but not miR-NC (Fig. 3a), suggesting either miR-589 or miR-1244 could induce the apoptosis of non-small cell lung cancer cells. As showed in Fig. 3b, the apoptosis rates of A549/DDP cells transfected with miR-1244 mimic, miR-589 mimic and control were 28.83 %, 38.78 versus 10.5 % (P < 0.05).

To further verify the miR-589 or miR-1244-induced cell apoptosis, we examined activation of the caspase-3, a hallmark of apoptosis. As showed by western blot (Fig. 3c), there is a significant band for cleaved caspase-3 in miR-589-transfected cells in comparison to the negative control at 24 h, indicating early events in the apoptosis pathway.

### miR-589 or miR-1244 reduces A549/DDP cell migration

To evaluate the impact of miR-589 or miR-1244 on the migration of the DDP-resistant cells, we performed cell wound healing assay. Interestingly, the migration rate of the transfected cells by either miR-589 or miR-1244 mimics was significantly reduced compared to miR-NC at both 24 h and 48 h (Fig. 3d, e), indicated that miR-589 or miR-1244 inhibits the cell mobility of A549/DDP cells.

### Molecular targets of miR-1244

**Fig. 4:**
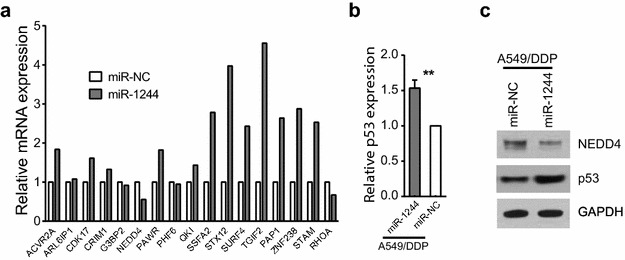
Expression levels of mRNA. **a** Expression levels of the predicted target genes in A549/DDP-miR-1244 cells and A549/DDP-miR-NC cells. **b** Expression level of TP53 in A549/DDP-miR-1244 cells and A549/DDP-miR-NC cells. The results are the average of triplicate experiments (**P < 0.05). **c** Western blot of NEDD4 and p53 expression in both A549/DDP-miR-1244 and A549/DDP-miR-NC cells. GAPDH was used as internal control

To gain insight into the possible molecular mechanisms underlying the striking difference in the expression of miRNA, especially miR-1244, between A549/DDP and A549 cells, we predicted the potential target of miR-1244 using miRDB (http://www.mirdb.org/miRDB/), Targetscan (http://www.targetscan.org/) and CLIP-Seq. Among hundreds of the target genes predicted, we selected the most likely target genes and validated their expressions by qPCR. Importantly, 13 out of 17 top genes were confirmed up-regulated in the A549/DDP cells transfected by miR-1244, while 4 target genes were confirmed down-regulated, particularly NEDD4 (Fig. 4a). As an E3 ligase, knockdown of NEDD4 was showed to increase basal TP53 levels, thus causing stronger TP53 responses to DNA damage [20], we therefore analyzed the expression level of TP53 by QPCR. Interestingly, TP53 was found over-expression in the A549/DDP upon transfection of miR-1244 (Fig. 4b), indicating TP53 is one of the potential targets of miR-1244. This observation was further confirmed at protein level by western blot. As showed in Fig. 4c, while NEDD4 was down-regulated, the translational product of TP53 was significantly induced in A549/DDP-miR-1244 compared to A549/DDP-miR-NC cell. These data imply p53 protein might be involved in the process of chemoresistance of tumor cells.

## Discussion

Despite the great advances in cancer therapy, chemo-resistance is still the daunting challenge for the treatment of advanced lung cancer. Accumulating studies suggest that miRNAs might be one of the molecular mechanisms of multidrug resistance in human cancers [21, 22]. For example, up-regulation of miR-451, miR-98 and miR-513a-3p increased cisplatin sensitivity in human lung adenocarcinoma cells [17, 18, 23], while miR-495 enhanced the sensitivity of NSCLC cells to platinum [24]. These studies highlighted the important role of miRNAs in regulating lung cancer DDP-resistance.

In this study, we observed that miR-1244 and miR-589 were significantly down-regulated in A549/DDP cell, while miR-224 and miR-182 were markedly up-regulated in A549/DDP cell. The corrected expression of miR-1244 and miR-589 increased the sensitivity of A549/DDP cells to cisplatin, and induced cell apoptosis. These results indicated that both miR-1244 and miR-589 play an important role in the development of cisplatin resistance in NSCLC.

To our best knowledge, this is the first study showing that miR-1244 was down-regulated upon acquisition of drug-resistance to cisplatin in A549 cell. MiR-1244 acts as a tumor suppressor in lung cancer by reducing its proliferation, survival and invasion, and its under-expression is highly associated with patients’ survival [25]. This could explain our observation that the rescued expression of miR-1244 could sensitize A549/DDP cells to DDP via inhibiting cell migration and/or increasing cell apoptosis, likely explaining the mechanism of miR-1244 in chemo-resistance of NSCLC.

Interestingly, miR-589 has been reported to repress epithelial-mesenchymal transition (EMT) in human peritoneal mesothelial cells [23]. While EMT is a critical event initiating cancer invasion and metastasis, and dysregulation of EMT could increases the drug-resistance to many therapeutic agents [26], thus perfect in line with our findings that overexpression of miR-589 sensitizes A549/DDP cells to cisplatin.

TP53 is a tumor suppressor gene and it is frequently mutated in a variety of human cancers including lung cancer, thus contributing to the acquisition and/or maintenance of drug resistance of malignant cancers. Our results showed over-expression of miR1244 in A549/DDP indirectly induced the expression of TP53 at both gene and protein levels. Therefore, down-regulated miR1244 in A549/DDP cell might contribute to the development of cisplatin-resistance via prohibiting TP53 regulated cell signaling pathway. These data underline the importance of miR-1244 in regulating the cisplatin resistance of NSCLC, and render a new angle toward target therapy of chemoresistance for cancer patients.

## Conclusion

The study indicates a crucial role of miR-1244 in regulating the cisplatin resistance of NSCLC. Further understanding of miR-1244-mediated signaling pathways may render a new angle toward target therapy of chemoresistance for cancer patients.
